# DMO-CAP inhibits influenza virus replication by activating heme oxygenase-1-mediated IFN response

**DOI:** 10.1186/s12985-019-1125-9

**Published:** 2019-02-20

**Authors:** Ming Zhong, Huiqiang Wang, Linlin Ma, Haiyan Yan, Shuo Wu, Zhengyi Gu, Yuhuan Li

**Affiliations:** 1 NHC Key Laboratory of Biotechnology of Antibiotics, Institute of Medicinal Biotechnology, Chinese Academy of Medical Science, Beijing, 100050 China; 2 Xinjiang Institute of Materia Medica, Urumqi, 830002 China; 3 Key Laboratory of Xinjiang Phytomedicine Resource and Utilization, Ministry of Education, Shihezi University, Shihezi, 832000 China; 4 Key Laboratory of Molecular Imaging of Shanghai Education Commission, Shanghai University of Medicine and Health Sciences, Shanghai, 201318 China

## Abstract

**Background:**

As a leading cause of respiratory disease, influenza A virus (IAV) infection remains a pandemic threat in annual seasonal outbreaks. Given the limitation of existing anti-influenza therapeutic drugs, development of new drugs is urgently required. Flavonoids extracted from *Artemisia rupestris* L. have an inhibitory effect on virus infections. Despite this fact, the antiviral properties of 6-demethoxy-4′-O-methylcapillarisin (DMO-CAP), one of such flavonoids, against the influenza virus have not been reported. Thus, the aim of this study is to investigate the anti-IAV virus efficacy and antiviral mechanism of DMO-CAP.

**Methods:**

The inhibitory activity of DMO-CAP against IAV was detected in vitro using viral titers by Western blot analysis, qRT-PCR, and immunofluorescence assays. The mechanism of DMO-CAP against influenza virus was analyzed by Western blot analysis, qRT-PCR, and luciferase assay.

**Results:**

DMO-CAP exhibits broad spectrum of antiviral activities against IAV in vitro. Mechanistically, DMO-CAP treatment induced the phosphorylation of p38 mitogen-activated protein kinase (MAPK), JNK MAPK, and ERK MAPK, which led to the activation of Nrf2/heme oxygenase-1 (HO-1) pathway. Then, the up-regulation of HO-1 expression activated the IFN response and induced the expression of IFN-stimulated genes, thereby leading to efficient anti-IAV effects.

**Conclusions:**

DMO-CAP inhibited IAV replication by activating HO-1-mediated IFN response. DMO-CAP may be a potential agent or supplement against IAV infection.

## Background

Influenza is one of the most common yet serious infectious diseases that represent a significant hazard to public health. Globally, annual epidemics cause 3 to 5 million cases of severe disease, millions of hospitalizations, and up to 650,000 deaths worldwide [1, 2]. The outbreak of avian influenza virus in recent years suggests that influenza still poses an ongoing and powerful threat to humans [3].

Although administration of vaccines seem a vital strategy for prophylaxis, the lag time between virus identification and vaccine distribution weakens its preventive effect. In the short time, antiviral therapy is the best option to control the spread of influenza. To date, licensed drugs in the clinic only include M2 ion-channel blockers (amantadine and rimantadine), neuraminidase inhibitors (oseltamivir and peramivir), and RNA-dependent RNA polymerase (RdRp) inhibitor (favipiravir [T705]) [4–6]. Lately, the US Food and Drug Administration has approved Xofluza™ (baloxavir marboxil) for the treatment of acute, uncomplicated influenza, or flu, in people 12 years old and older. Xofluza is a first-in-class, single-dose oral medicine with a novel proposed mechanism of action that inhibits polymerase acidic endonuclease. It exhibited efficient activities against a wide range of influenza viral infection, including oseltamivir-resistant and avian strains (H7N9 and H5N1) in nonclinical studies [7, 8]. However, the rapid emergence of drug-resistant viral mutants restricts the utilization of these drugs [9]. Thus, a safer and more effective anti-IAV drugs must be developed.

In contrast to the virus, host factors do not change quickly. Therefore, overpowering influenza by targeting host factors involved in viral replication is a potentially effective strategy. Such a strategy may weaken the virus’ ability to evolve resistance [10]. Heme oxygenase-1 (HO-1) is an inducible enzyme that degrades pro-oxidant heme into equimolar quantities of carbon monoxide (CO), iron, and biliverdin [11]. HO-1 is an effective cytoprotection because of its antioxidant and anti-inflammatory properties [12]. In addition, HO-1 regulates innate immunity and autoimmunity by modulating IFN-β production, which can control viral infections, such as human immunodeficiency virus, hepatitis B virus, hepatitis C virus, Ebola virus, RSV, dengue, and influenza A virus (IAV) [13–17]. Specifically, Ma et al. found that YZH-106, a rupestonic acid derivative, presented effective anti-IAV activity by activating HO-1-mediated type I IFN response [16]. In 2012, Cummins et al. demonstrated that HO-1 can regulate the immune response to influenza virus infection and vaccination in aged mice [17].

In this study, we first presented that 6-demethoxy-4′-O-methylcapillarisin (DMO-CAP), a flavonoid derivative of *Artemisia rupestris* L., exerts a wide spectrum of anti-IAV activity. IAV replication was inhibited after the activation of HO-1-mediated type I IFN signal pathway by DMO-CAP.

## Methods

### Compounds

DMO-CAP is a separation and purification of the 50% ethanol-eluted fractions extracted from *Artemisia rupestirs L.* The compound structure was confirmed with LC-HRMS and MS spectra [18]. In this study, 67 mM stock solutions of DMO-CAP were prepared in dimethyl sulfoxide (DMSO, Sigma-Aldrich, Carlsbad, CA). Oseltamivir carboxylate (OC, Medchem, Princeton, NJ, USA), amantadine hydrochloride (AH, sigma-Aldrich, St Louis, MO, USA) and ribavirin (RBV, Sigma-Aldrich, Carlsbad, CA) were used as reference compounds. Furthermore, 20 mM stock solutions of OC were prepared in DMSO. 20 mM stock solutions of RBV were prepared in culture medium. These drugs were configured to the essential experimental concentrations.

### Cell lines, viral strains and viral infection

Madin-Darby canine kidney (MDCK) cells were purchased from America Type Culture Collection (ATCC) and cultured in minimum essential medium (MEM; Invitrogen, Carlsbad, CA) comprised 10% fetal bovine serum (Gibco, Grand Island, NY), 1% antibiotics (100 U/ml penicillin and 100 mg/ml streptomycin) (Invitrogen, Carlsbad, CA). Mouse macrophage RAW264.7 cells were obtained from Cell Resource Center at Institute of Basic Medical Sciences, Chinese Academy of Medical Sciences, Beijing, China, cultivated in Dulbecco’s Modified Eagle Medium (DMEM, Invitrogen), containing 10% FBS and 1% antibiotics. Human embryonal kidney (HEK293T-17) cells were purchased from the Cell Culture Center of Peking Union Medical College and cultured in Dulbecco’s Modified Eagle Medium (Invitrogen, Carlsbad, CA, USA) supplemented with 10% FBS and 1% antibiotics at 37 °C in a 5% CO_2_ incubator.

Influenza strain A/Fort Monmouth/1/1947 (H1N1) was purchased from America Type Culture Collection (ATCC). Clinical isolated A/Wuhan/359/1995 (H3N2), A/LiaoningZhengxin/1109/2010 (H1N1, oseltamivir resistant strain), and A/HunanZhuhui/1222/2010 (H3N2, amantadine resistant strain) were kindly provided by Yuelong Shu, Ph.D., Professor, the Institute for Viral Disease Control and Prevention, China Centers for Disease Control and Prevention. IAV strains were prepared by propagating in 10-day-old embryonated chicken eggs for 72 h.

For infections of MDCK cells, cells were washed with PBS and infected with influenza virus at indicated multiplicity of infection in serum-free medium for 2 h at 37 °C. accompanying the supernatant was removed and replaced by maintenance medium supplemented with 2 μg ml^− 1^ TPCK-treated trypsin (Worthington, Lakewood, Colorado, USA) and 0.08% BSA (Beijing Yuan Heng Golden Horse biological technology development Co., Ltd., China). For RAW264.7 cells, the maintenance medium was supplemented with 2% FBS.

### Cytotoxicity test

The cytotoxicity effects of compounds on cells were evaluated by CCK Kit (TransGen Biotech, Beijing, China) [19]. Briefly, MDCK and RAW264.7 cells were cultured in 96-well plates and different concentrations of DMO-CAP were applied in two-fold dilution for 48 h. Then, 10 μl CCK solution was added to each well. After incubating at 37 °C for 1 h, the plates were detected by scanning absorbance at 450 nm on Enspire (Perkin Elmer, Waltham, MA). The 50% toxicity concentration (TC_50_) of DMO-CAP was calculated by Reed and Muench method [20].

### Cytopathic effect (CPE) assay

MDCK cells infected with influenza virus at 100TCID_50_ for 2 h, following the unbound viruses were removed and treated with or without the tested compounds for 48 h [19]. Then, the 50% inhibitory concentration (IC_50_) was calculated based on Reed and Muench method and the selectivity index (SI) of compounds was calculated as the ratio of TC_50_/IC_50_ [21].

### Western blot assay

For whole-cell extract preparation, the cells were lysed in M-PER mammalian protein extraction reagent containing halt protease inhibitor cocktail (Thermo Fisher Scientific, Waltham, MA, USA), and the nuclear and cytosolic extracts were prepared using nuclear and cytoplasmic extraction kit (Beyotime, Beijing, China).

Cell lysate was subjected to 10%SDS-PAGE gel. Proteins were transferred onto a 0.2 μM PVDF membrane (Thermo Fischer Scientific). Membranes were blocked with 5% milk for 2 h and incubated overnight at 4 °C with specific primary antibody. After a standard washing, membranes were incubated with horse radish peroxidase (HRP)-labeled secondary antibody. The assay developed using a chemiluminescent substrate. The primary antibodies used in this study included antibodies against p38, P-P38, ERK, P-ERK, JNK/SAPK, P-JNK/SAPK, HO-1, Nrf2, PKR, IFIT1, OAS1, histone H3, β-actin (Cell Signaling Technology, Beverly, MA, USA) and IAV M2, NS1 (Santa Cruz, Dallas, Texas, USA). The goat anti-rabbit and anti-mouse HRP-labeled antibodies were obtained from Cell Signaling Technology.

### Quantitative real-time RT-PCR

Total cellular RNAs were extracted using RNeasy Mini Kit (Qiagen, USA). IAV M2 mRNA, IFN-α mRNA, and IFN-β mRNA were measured by One-Step qRT-PCR using primers reported as Table 1 showed. GAPDH mRNA served as internal control to normalize tested mRNAs. The using reaction system and conditions reported as previously [16].

**Table 1:** Oligonucleotides used for real-time RT-PCR

OLIGONUCLEOTIDE	SEQUENCE (5′–3′)
5′ M2 (INFLUENZA)	GACCRATCCTGTCACCTCTGAC
3′ M2 (INFLUENZA)	GGGCATTYTGGACAAAKCGTCTACG
5’ IFN-β (M)	AGCTCCAAGAAAGGACGAACAT
3’ IFN-β (M)	GCCCTGTAGGTGAGGTTGATCT
5’ IFN-α (M)	CCTGTGTGATGCAACAGGTC
3’ IFN-α (M)	TCACTCCTCCTTGCTCAATC
5’ GAPDH (M)	CTCTGGAAAGCTGTGGCGTGATG
3’ GAPDH (M)	ATGCCAGTGAGCTTCCCGTTCAG
5’ GAPDH (D)	AGTCAAGGCTGAGAACGGGAAACT
3’ GAPDH (D)	TCCACAACATACTCAGCACCAGCA

### Immunofluorescence assay

MDCK cells infected IAV were mock-treated or treated with the indicated concentrations of DMO-CAP for 24 h. The cells were fastened with PBS containing 4% paraformaldehyde followed by incubation with 0.1% Triton X-100 for 20 min. Cells were then blocked and incubated with an antibody against IAV M2 (Santa Cruz, Dallas, T). Bound primary antibody was visualized by Alexa Fluor 488-conjugated secondary antibody (Invitrogen). Cell nuclei were stained with DAPI (Beyotime, Shanghai, China). Pictures were taken with an Olympus TH4–200 microscope [19].

### Luciferase assay

Luciferase reporter genes activities responded to Nrf2, NF-κB and AP-1 were detected with Dual-Glo Luciferase Assay System (Promega, Mullion, WI, USA). HEK293T-17 cells seeded in 12-well plate were cotransfected with pGL4.37[luc2P/ARE/Hygro] (Promega)/pAP-1-Luc/pNF-κB-Luc (provided by Professor Jian-ping Ye at Pennington Biomedical Research Center, Louisiana State University, LA, USA) expressing firefly luciferase and pRL-SV40 vector (Promega) expressing renilla luciferase in a 10:1 mass ratio. After the infection of IAV and treatment with DMO-CAP for 24 h, Lysis cells and collect supernatants at 4 °C, 12,000 rpm. Then 10 μl sample supernatant and 40 μl of luciferase reagent was added to white 96-well plate and the firefly luminescence was measured after 10 min on Enspire. Then, 40 μl of Stop & Glo Reagent was added and renilla luminescence was measured in the same plate after 10 min. Luciferase activities were calculated by the ratio of the firefly luminescence to the renilla luminescence.

### Statistical analyses

All data are given as the mean ± standard deviation (SD). Two groups were compared by student’s-test, more groups were compared by one-way ANOVA using GraphPad Prism6.0 software. Differences with the *P* value of < 0.05, 0.01 and 0.001 were considered statistically significant.

## Results

### Cytotoxicity and antiviral activity of DMO-CAP in vitro

**Fig. 1:**
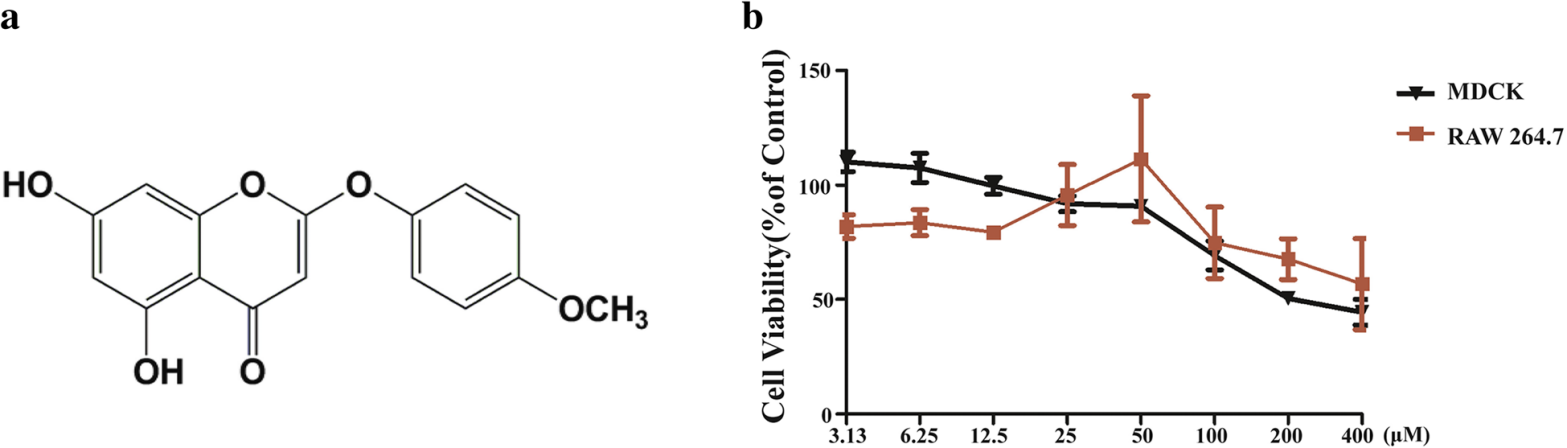
The structure and cytotoxicity of compound DMO-CAP. **a** The structure of DMO-CAP. **b** The viabilities of DMO-CAP on MDCK and RAW264.7 cells was measured by CCK assay

DMO-CAP (Fig. 1a), a flavonoid monomer, was obtained from 50% ethanol-eluted fractions separated and purified from *Artemisia rupestris* L*.* [18]. To determine the antiviral activity of DMO-CAP, we initially studied the cytotoxicity of DMO-CAP in MDCK and RAW 264.7 cells cells by CCK assay. The TC_50_ value of DMO-CAP is 200 and 400 μM in MDCK and RAW 264.7 cells, respectively, with an incubation time of 48 h (Fig. 1b). Thus, the highest concentration of DMO-CAP was set as 50 μM in the following antiviral assays, which confers minimal to no cellular cytotoxicity.

**Fig. 2:**
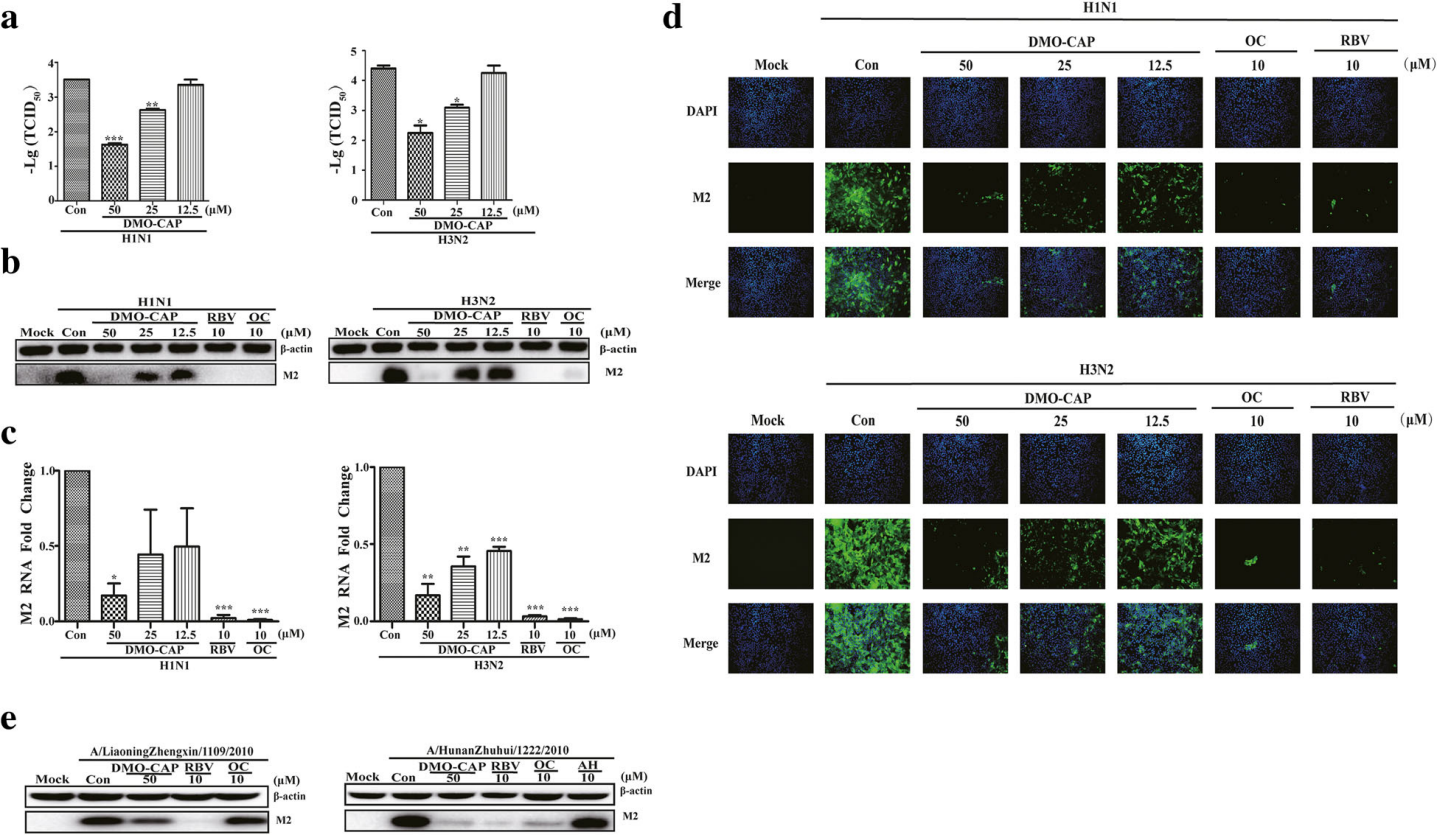
Antiviral activity of DMO-CAP against IAV. **a** Antiviral activity of DMO-CAP in RAW264.7 cells against A/Fort Monmouth/1/1947 (H1N1) and IAV A/Wuhan/359/1995 (H3N2) was tested by viral titers assay. **b** and **c** DMO-CAP reduced the expression of M2 RNA and protein in MDCK cells by one-step qRT-PCR assay and Western blot assay. **d** DMO-CAP reduced the expression of M2 protein by immunofluorescence. **e** DMO-CAP reduced the expression of M2 protein of influenza resistant strains by Western blot assay. Mock: normal cells without treatment; H1N1: cells were infected with IAV A/FM1/1947 at 0.01MOI; H3N2: cells were infected with IAV A/Wuhan/359/1995 (H3N2) at 0.01MOI. Con: cells were infected pathognomonic viral strains and treated with equal amounts of DMSO or DMO-CAP. The experiments were performed in triplicate and the data represents mean ± SD. ***P < 0.001, **P < 0.01, *P < 0.05 versus Con

Through CPE assay, the selectivity index (SI) of DMO-CAP against A/FortMonmouth/1/1947 (H1N1) and A/Wuhan/359/1995 (H3N2) ranges from 5.2 to 7.20 (Table 2). Meanwhile, the antiviral efficacy of DMO-CAP was also tested using viral titers reduction assay. We observed a dose-dependent reduction in viral titers when the cells were treated with DMO-CAP after IAV infection (Fig. 2a). In addition, we evaluated the inhibition ability of DMO-CAP against IAV by Western blot and qRT-PCR analysis. DMO-CAP dose-dependently reduced the amounts of IAV M2 protein and RNA in vitro (Fig. 2 b and c). To further confirm that DMO-CAP inhibited viral protein synthesis, we analyzed the expression of the viral M2 protein through indirect immunofluorescence assay. In Fig. 2d, DMO-CAP exhibited a dose-dependent inhibition of the M2 protein expression in vitro. In addition, as shown in Fig. 2e, DMO-CAP can inhibit the expression of M2 protein on other influenza strains, including A/LiaoningZhengxin/1109/2010 (H1N1, oseltamivir resistant strain) and A/HunanZhuhui/1222/2010 (H3N2, amantadine resistant strain). Overall, DMO-CAP exhibited a wide range of effective antiviral activities against IAV infection.

**Table 2:** Inhibitory activities of compounds against influenza strains

		A/FortMonmouth/1/1947		A/Wuhan/359/1995	
	TC_50_ (μM)	IC_50_ (μM)	SI	IC_50_ (μM)	SI
DMO-CAP	223 ± 0.41	31.78 ± 0.51	7.02	42.91 ± 0.60	5.20
OC	> 487 ± 0.12	0.56 ± 0.09	> 869.64	0.69 ± 0.24	> 705.80
RBV	> 890 ± 0.13	1.02 ± 0.12	> 872.55	1.97 ± 0.22	> 451.78

### DMO-CAP inhibits influenza virus replication through up-regulation of HO-1 expression

HO-1, an inducible enzyme expressed in the irritant of physical and chemical stresses, has proven its cytoprotectant, antioxidant, and antiapoptotic properties against many diseases [22, 23]. DMO-CAP, identified as flavonoids, a classical medicinal ingredient, has been demonstrated to have anti-inflammatory and antioxidant activities, which are partly similar to the function of HO-1 [18]. Thus, we aimed to determine whether the anti-IAV activity of DMO-CAP was related to HO-1.

**Fig. 3:**
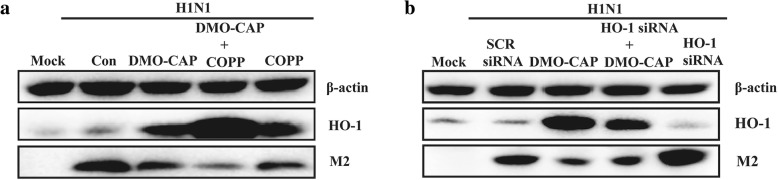
DMO-CAP inhibited IAV replication through up-regulating HO-1 expression. **a** HEK293T-17 cells were infected with IAV A/FM1/1947 (0.2 MOI) and then treated with DMO-CAP (50 μM)、CoPP (2 μM) together with DMO-CAP (50 μM) and CoPP (2 μM) for 24 h. **b** HEK293T-17 cells were transfected with HO-1 siRNA or SCR siRNA for 24 h and then infected with IAV A/FM1/1947 (0.2 MOI) in the absence or presence of DMO-CAP for 24 h. The expression of M2 and HO-1 proteins were analyzed by Western blot

To verify the hypothesis, we used cobaltic protoporphyrin IX chloride (CoPP), a potent HO-1 inducer, to confirm the antiviral effect of HO-1 on IAV infection. As shown in Fig. 3a, both DMO-CAP and CoPP can up-regulate the expression of HO-1 and inhibit the replication of IAV. In addition, the combined treatment of DMO-CAP and CoPP can synergistically up-regulate the expression of HO-1 and have cooperative effects on inhibiting IAV replication in vitro. Then, the cells were transfected with HO-1 siRNA or scrambled (SCR) siRNA and infected with IAV A/FM1/1947. As shown in Fig. 3b, compared with SCR siRNA, HO-1 siRNA treatment partially enhanced IAV replication and reversed the antiviral effect of DMO-CAP to some extent. Up-regulation of HO-1 is at least partly required for DMO-CAP to inhibit the replication of IAV.

### DMO-CAP activates the interferon response by stimulating the Nrf2/ARE pathway to up-regulate the expression of HO-1

**Fig. 4:**
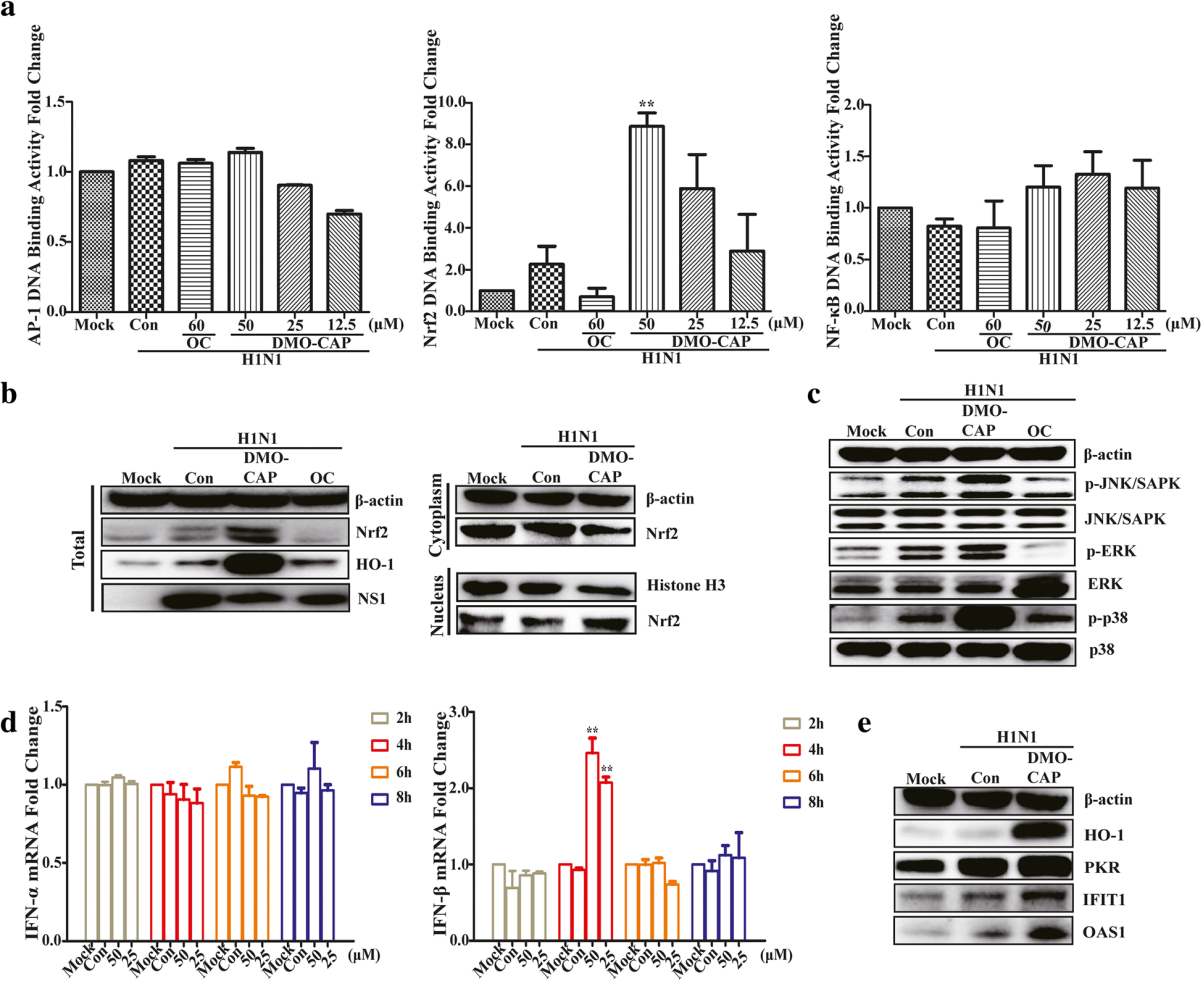
DMO-CAP activates the interferon response by stimulating the Nrf2/ARE pathway to up-regulate the expression of HO-1. **a** Nrf2 DNA binding activity was analyzed in HEK293T-17 cells co-transfected with pGL4.37 [luc2P/ARE/Hygro]/pAP-1-Luc/pNF-κB-Luc and pRL-SV40 vector, the results were presented as Nrf2 DNA binding activity relative to its basal levels in mock 293 T. **P < 0.01 versus Mock. **b** DMO-CAP promoted Nrf2 nuclear transcription. RAW264.7 cells were infected with IAV A/FM1/1947 (0.2 MOI) for 2 h and treated with 50 μM DMO-CAP for another 3 h. The total amount of cellular, cytoplasmic and nuclear Nrf2 protein were analyzed by Western blot. **c** DMO-CAP activated the phosphorylation of p38 MAPK, JNK MAPK and ERK MAPK. RAW264.7 cells were infected with IAV A/FM1/1947 (0.2 MOI) and treated with 50μΜ DMO-CAP for 15 min and then phospho-p38、phospho-JNK and phospho-ERK proteins were valued by Western blot. **d** RAW264.7 cells were infected with IAV A/FM1/1947 (0.2 MOI) for 2 h, followed by treating with or without DMO-CAP (50μΜ or 25μΜ). The mRNA level of IFN-α and IFN-β were detected by qRT-PCR assay. **P < 0.01 versus Mock. **e** RAW264.7 cells were infected with IAV A/FM1/1947 (0.2 MOI) for 2 h and the protein levels of ISGs were measured by Western blot after treatment with DMO-CAP for 24 h

HO-1 expression is up-regulated not only by its substrate, heme, but also by various non-heme inducers, such as heat shock, inflammatory cytokines, endotoxin, and oxidative stress [24, 25]. Antioxidant responsive element (ARE), one of transcription factors binding to HO-1 gene and Nrf2/ARE complex, had been found that could regulate HO-1 expression [26, 27]. To detect the upstream signaling pathway of HO-1 induced by DMO-CAP, luciferase reporter genes activities responded to Nrf2, NF-κB and AP-1, three typical nuclear transcription factors associated with the transactivation of HO-1 expression, were detected with Dual-Glo Luciferase Assay System. As shown in Fig. 4a, ARE-driven luciferase activity responding to Nrf2 binding was induced by DMO-CAP in a dose-dependent manner; whereas, no effect was observed on the other two transcription factors. Furthermore, the result of nuclear/cytosol fractionation analysis shows that nuclear Nrf2 accumulation was induced by DMO-CAP within 3 h in IAV-infected RAW 264.7 cells, whereas HO-1 protein expression increased (Fig. 4b).

Mitogen-activated protein kinase (MAPK) signaling pathways are composed of three subfamilies, including ERK MAPK, JNK MAPK, and p38 MAPK; the MAPK signaling pathway regulates important cellular processes of defense pathogen invasion [28, 29]. Nrf2-mediated HO-1 expression is reportedly related to the activation of MAPK signaling pathway [30]. To clarify the relationship between the up-regulation of Nrf2 and MAPKs after DMO-CAP treatment, we detected the phosphorylation level of MAPKs. As shown in Fig. 4c, DMO-CAP can stimulate the phosphorylation levels of ERK MAPK, JNK MAPK, and p38 MAPK in 15 min. DMO-CAP can up-regulate the Nrf2-mediated HO-1 expression by MAPK pathways in the RAW264.7 cells.

HO-1 has been known to regulate IFN production and play an important role in suppressing viral replications, including IAV [31, 32]. Hence, to determine whether HO-1 up-regulation by DMO-CAP activates antiviral IFN response in the case of IAV infection, we studied the effect of DMO-CAP on the expressions of IFN-α/β and ISGs. As indicated in Fig. 4d, we found that DMO-CAP enhanced the mRNA expression of IFN-β at 4 h post infection, whereas no effect was observed on the mRNA expression of IFN-α. We also found that DMO-CAP treatment induced the expression of ISGs, such as interferon-induced protein with tetratricopeptide repeats 1 (IFIT1), double-stranded RNA-dependent protein kinase (PKR), and 2′-5′-oligoadenylate synthetase 1 (Fig. 4e). We discovered that HO-1 up-regulation by DMO-CAP activates antiviral IFN response, followed by induction of ISG protein in IAV-infected RAW264.7 cells.

## Discussion

HO-1, a stress-induced and cytoprotective enzyme expressed in most cell types that catalyze heme metabolism into CO, iron, and biliverdin, has been proven to play a vital role in modulating immune responses [33–35]. HO-1 has been known to inhibit viral infections through regulation of immune responses, such as CoPP decreased RSV replication through increasing the production of HO-1-modulated IFN-α/β in vivo [36]. Consistent with these findings, Ma et al. found that HO-1 overexpression stimulated significant up-regulation of IFN-α/β and inhibited replication of IAV [16]. This study is the first to demonstrate the anti-IAV activity and antiviral mechanism of DMO-CAP. The study results showed that induction of HO-1 expression by DMO-CAP treatment enhanced IFN-β expression in the RAW264.7 cells. Thus, DMO-CAP can dose-dependently inhibit replication of IAV.

**Fig. 5:**
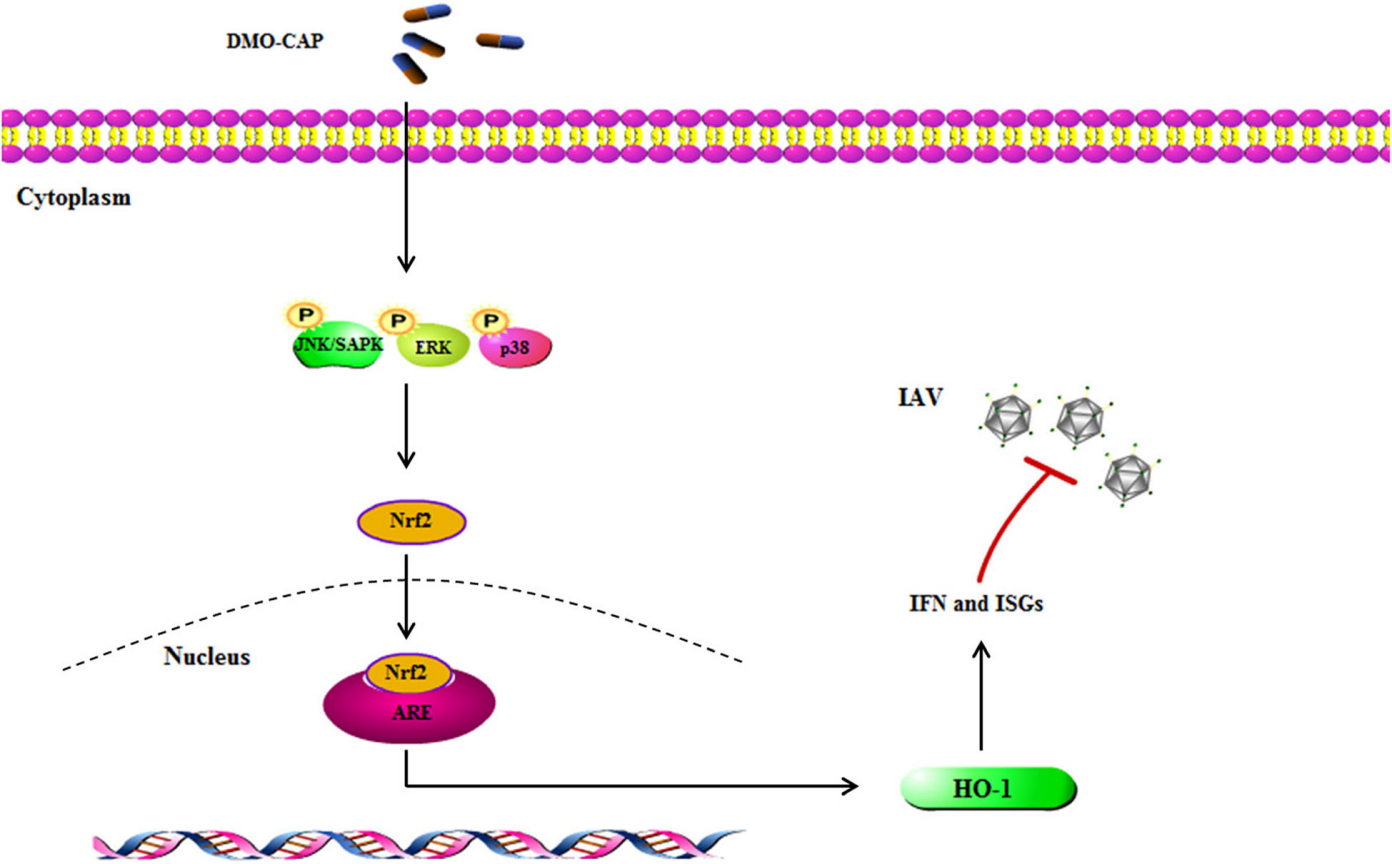
Schematic showing that DMO-CAP inhibits influenza virus replication. DMO-CAP activated the MAPK pathways, thereby leading to Nrf2 expression and subsequent activation of HO-1 gene expression, as well as the up-regulation HO-1 activities host cellular type I IFN response with induction of ISGs expression, which finally leads to inhibition of influenza virus replication

The innate immune system plays an important role in protection against IAV infections. HO-1-mediated inhibition of viral replication is at least partly associated with IFN-α/β induction, which increased the expression of ISG genes, such as IFIT1, OAS, and PKR [37]. In this study, DMO-CAP activated the MAPK pathways, thereby leading to Nrf2 expression and subsequent activation of HO-1 gene expression, as well as the up-regulation HO-1 activities host cellular type I IFN response with induction of ISGs expression (Fig. 5). Thus, the broad spectra of antiviral activities of DMO-CAP are much likely to be associated with the induction of ISGs.

Flavonoids have a variety of biological activities, such as anti-inflammatory and antioxidant properties. Flavonoids play an important role in regulating virus replication by inhibiting oxidative stress and inflammation [38, 39]. DMO-CAP, a flavonoid monomer, was verified to be effective against influenza virus activity in this study. Our study found that DMO-CAP inhibits IAV replication at least in part by up-regulating the expression of HO-1, thereby activating interferon response (Fig. 5). Therefore, further studies are needed to clarify whether DMO-CAP has other mechanisms to inhibit influenza virus replication.

Our research is the first to report the anti-IAV mechanism of DMO-CAP, and our findings provide a new clue for the development of an anti-IAV drug, which induces HO-1 expression. However, many questions remain, e.g., whether DMO-CAP is an effective antiviral therapy against IAV without adverse toxic effects in vivo.

## Conclusion

In this study, it was found that DMO-CAP treatment induced the phosphorylation of p38 MAPK, JNK MAPK, and ERK MAPK, which led to the activation of Nrf2/heme oxygenase-1 (HO-1) pathway. Then, the up-regulation of HO-1 expression activated the IFN response and induced the expression of IFN-stimulated genes, thereby leading to efficient anti-IAV effects. Taken together, our data demonstrated that DMO-CAP may be a potential agent or supplement against IAV infection.

## Abbreviations

AP-1: Activator protein-1
ARE: Antioxidant response element
CoPP: Cobalt protoporphyrin
CPE: Cytopathic effect
IAV: Influenza A virus
IC_50_: 50% inhibitory concentration
IFIT1: IFN-induced protein with tetratricopeptide repeats 1
ISGs: IFN-stimulated genes
MAPK: Mitogen-activated protein kinase
NF-κB: Nuclear factor kappa B
Nrf2: Nuclear factor-erythroid 2-related factor 2
OAS1: 2′-5′-Oligoadenylate synthetase 1
PKR: double-stranded RNA-dependent protein kinase
SI: Selectivity index
TC_50_: 50% toxicity concentration
TCID_50_: 50% tissue culture infective doses
